# Antimicrobial and Biofilm-Preventing Activity of *l*-Borneol Possessing 2(5*H*)-Furanone Derivative F131 against *S. aureus*—*C. albicans* Mixed Cultures

**DOI:** 10.3390/pathogens12010026

**Published:** 2022-12-23

**Authors:** Rand Sulaiman, Elena Trizna, Alena Kolesnikova, Alsu Khabibrakhmanova, Almira Kurbangalieva, Mikhail Bogachev, Airat Kayumov

**Affiliations:** 1 Laboratory of Molecular Genetics of Microorganisms, Institute of Fundamental Medicine and Biology, Kazan Federal University, 420008 Kazan, Russia; 2 Biofunctional Chemistry Laboratory, Alexander Butlerov Institute of Chemistry, Kazan Federal University, 420008 Kazan, Russia; 3 Biomedical Engineering Research Centre, St. Petersburg Electrotechnical University, 197022 St. Petersburg, Russia

**Keywords:** antimicrobial resistance (AMR), mixed biofilms, synergy, 2(5*H*)-furanones, sulfones

## Abstract

*Candida albicans* and *Staphylococcus aureus* are human pathogens that are able to form mixed biofilms on the surface of mucous membranes, implants and catheters. In biofilms, these pathogens have increased resistance to antimicrobials, leading to extreme difficulties in the treatment of mixed infections. The growing frequency of mixed infections caused by *S. aureus* and *C. albicans* requires either the development of new antimicrobials or the proposal of alternative approaches to increase the efficiency of conventional ones. Here, we show the antimicrobial, biofilm-preventing and biofilm-eradicating activity of 2(5*H*)-furanone derivative **F131,** containing an *l*-borneol fragment against *S. aureus–C. albicans* mixed biofilms. Furanone **F131** is also capable of inhibiting the formation of monospecies and mixed biofilms by *S. aureus* and *C. albicans*. The minimal biofilm-prevention concentration (MBPC) of this compound was 8–16 μg/mL for *S. aureus* and *C. albicans* mono- and two-species biofilms. While the compound demonstrates slightly lower activity compared to conventional antimicrobials (gentamicin, amikacin, fluconazole, terbinafine and benzalkonium chloride), **F131** also increases the antimicrobial activity of fluconazole–gentamicin and benzalkonium chloride against mixed biofilms of *S. aureus–C. albicans*, thus reducing MBPC of fluconazole–gentamicin by 4–16 times and benzalkonium chloride twofold. **F131** does not affect the transcription of the MDR1, CDR1 and CDR2 genes, thus suggesting a low risk of micromycete resistance to this compound. Altogether, combined use of antibiotics with a **F131** could be a promising option to reduce the concentration of fluconazole used in antiseptic compositions and reduce the toxic effect of benzalkonium chloride and gentamicin. This makes them an attractive starting point for the development of alternative antimicrobials for the treatment of skin infections caused by *S. aureus–C. albicans* mixed biofilms.

## 1. Introduction

*Staphylococcus aureus* is a common opportunistic microorganism causing various infectious diseases in human and animals. Together with various virulence factors allowing host colonization, tissue invasion, and host immune system evasion, this bacterium also possesses different tools for overcoming the treatment with antimicrobials [1,2], that makes *S. aureus* one of the most dangerous nosocomial pathogens [3,4]. Thus, based on its susceptibility to antibiotics, *S. aureus* is classified into methicillin-susceptible *S. aureus* (MSSA) and methicillin-resistant *S. aureus* (MRSA) [5,6]. In the last decade, MRSA quickly became the most common drug-resistant pathogen identified in many parts of the world [7,8,9].

The biofilm formation by *S. aureus* on various immunogenic and non-immunogenic surfaces of the human body represents an additional challenge in medicine [10]. While in the biofilm, cells intensively produce factors of virulence, induce horizontal genes transfers and remain inaccessible for antimicrobials and the immune system of the host due to a physical barrier formed by an extracellular matrix [11,12,13,14,15]. Consequently, rapid development of multiple resistances to antimicrobials occurs, as well as the acute infection transforms to persistent, chronic and recurrent infection [16,17,18].

In immune-compromised patients, *S. aureus* is often associated with other nosocomial pathogens, such as *E. coli, P. aeruginosa, K. pneumoniae* and *C. albicans* [19,20,21,22,23]. The microscopic fungi *C. albicans* colonizes many niches of the human body, and has a wide range of tools to survive under the conditions of the treatment, including morphological transition between yeast and hyphal forms, adhesins expression, biofilm formation and secretion of hydrolytic enzymes [24]. Moreover, in response to treatment, the increased synthesis of membrane efflux transporters Cdr2p and Mdr1p is induced, in addition to constitutively expressed Cdr1p, and leads to extremely high resistance development [25,26]. In humans, it causes various infections of the mucous membranes, skin, nails and the gastrointestinal tract [27,28,29,30,31,32].

*C. albicans* also often forms biofilms, and their emergence is positively correlated with increased virulence and higher patient mortality [33,34]. In mixed communities, *S. aureus* and *C. albicans* form rigid biofilms on medical implants and catheters [35] in which the fungi provide a scaffold for the bacteria [36,37]. Thus, *S. aureus* exhibits high affinity for the hyphae shape of *C. albicans* because these species are able to interlock and interact synergistically, forming a dense and architecturally complex biofilm [37,38]. For example, the onset of oral candidiasis can lead to systemic staphylococcal infection resulting from a thick matrix of mixed biofilm formed on the tongue tissue [39,40]. In vitro studies have shown that in *C. albicans*–*S. aureus* biofilm, microorganisms exhibit an increased resistance due to the high content of polysaccharides synthesized by *C. albicans*, which provide low permeability of antimicrobials into the biofilm [21]. As a result, the complexity of these polymicrobial infections creates an additional challenge for finding effective treatment strategies, and the development of new approaches to treat polymicrobial communities remains the most urgent task of medical microbiology.

Among various compounds with antibiofilm activity, 2(5*H*)-furanone derivatives have been suggested [41,42,43]. These compounds are the natural tool of the red algae *Delisea pulchra*, suppressing their biofouling [44,45,46]. Various furanones can be either synthesized in natural conditions by various microorganisms and plants or obtained chemically [47,48]. In cells, furanones are involved in intraspecific and interspecific signaling and communication, and also they act as attractants, pheromones and antimicrobials [49]. For natural furanones, high antimicrobial activity was shown, as well as the ability to reduce the formation of biofilms by *E. coli, P. aeruginosa, B. subtilis*, *B. cereus*, *Staphylococcus* spp. and *C. albicans* [41,43,44,50,51,52,53,54,55]. Beyond their ability to prevent the formation of biofilms, some 2(5*H*)-furanone derivatives also exhibit antibacterial activity against *S. aureus* [41,56] and *S. epidermidis* [57]. In addition, some studies have shown that furanones are able to increase microbial susceptibility to antibiotics [41,53,58,59]. In addition, non-toxic concentrations of furanones can increase the efficiency of fluconazole and terbinafine against resistant strains of *C. albicans* [53]. The 2(5*H*)-furanone derivative possessing menthol moiety and sulfonyl group has been reported to enhance the activity of some antimicrobials against *S. aureus* and *C. albicans* [41,53]. Here, we aim to evaluate the ability of novel 2(5*H*)-furanone derivative **F131,** possessing two pharmacophores (2(5*H*)-furanone cycle and *l*-borneol moiety) to exhibit an antimicrobial and biofilm-preventing activity and to potentiate conventional antibiotics and antifungal agents against mixed cultures of *S. aureus* and *C. albicans*.

## 2. Materials and Methods

### 2.1. Chemistry (General Information)

The 3,4-Dichloro-5-hydroxyfuran-2(5*H*)-one (mucochloric acid, **1**) (Vekton, Russia) was commercially available and was further recrystallized from water, mp 127 °C. The (1*S*,2*R*,4*S)*-(–)-Borneol and 4-chlorothiophenol (Acros Organics) were used without further purification. All solvents were purified and distilled by standard procedures. IR spectra were recorded on a Bruker Tensor-27 spectrometer fitted with a Pike MIRacle ATR accessory (diamond/ZnSe crystal plate). IR spectra were recorded of solids with characteristic absorption wavenumbers (*ν*_max_) reported in cm^−1^. NMR spectra were measured on a Bruker Avance III 400 spectrometer at 400.17 MHz (^1^H) and 100.62 MHz (^13^C) at 20 °C in the deuterated chloroform. The chemical shifts (δ) are expressed in parts per million (ppm) and are calibrated using residual undeuterated solvent peak as the internal reference (CDCl_3_: δ_H_ 7.26, δ_C_ 77.16). All coupling constants (*J*) are reported in Hertz (Hz) and multiplicities are indicated as: s (singlet), m (multiplet). High-resolution mass spectra (HRMS) were obtained by electrospray ionisation (ESI) with positive (+) ion detection on a Bruker micrOTOF–III quadrupole time-of-flight mass spectrometer. Analytical thin layer chromatography (TLC) was carried out on Sorbfil PTLC-AF-A-UF plates using dichloromethane as the eluent and UV light as the visualizing agent. The melting points were measured on an OptiMelt Stanford Research Systems MPA100 automated melting point apparatus and were not corrected. Optical rotations were measured on a Perkin-Elmer model 341 polarimeter at λ 589 nm and at 20 °C in chloroform (concentration *c* is given as g/100 mL).

### 2.2. Synthesis

The 3,4-Dichloro-5(*S*)-[(1*S*,2*R*,4*S*)-1,7,7-trimethylbicyclo[2.2.1]heptan-2-yloxy]-2(5*H*)-furanone (**2a**) was synthesized according to the known method [52,60].

***5(S)-3-Chloro-4-[(4-chlorophenyl)sulfanyl]-5-[(1S,2R,4S)-1,7,7-trimethylbicyclo[2.2.1]-heptan-2-yloxy]-2(5H)-furanone****(**3**)*. To a solution of furanone **2a** (0.40 g, 1.3 mmol) in dichloromethane (20 mL) with intense stirring was added dropwise a solution of 4-chlorothiophenol (0.19 g, 1.3 mmol) in dichloromethane (3 mL), and a solution of triethylamine (0.18 mL, 1.3 mmol) in dichloromethane (1 mL). The reaction mixture was stirred at room temperature for 2 h (monitored by TLC). After the completion of the reaction, the mixture was washed with water (100 mL), the organic layer was evaporated to dryness. The obtained solid residue was recrystallized from hexane to afford thioether **3**. Colorless solid (0.38 g, 71%); mp 128 °C; *R_f_* 0.61; [α]${}_{D}^{20}$ = –49.3 (CHCl_3_, *c* = 1.0). IR (ATR) ν_max_ 2979, 2949, 2882 (C–H), 1776 (C=O), 1599, 1477, 1455 (C=C_arom_) cm^−1^. ^1^H NMR (CDCl_3_, 400 MHz) *δ* 7.50, 7.39 (4H, AA′BB′, ^3^*J*_AB_ = ^3^*J*_A′B′_ = 8.2, ^4^*J*_AA′_ = ^4^*J*_BB′_ = 2.2, ^5^*J*_AB′_ = ^5^*J*_A′B_ = 0.3 Hz, H_arom_), 5.73 (1H, s, H-5), 3.77–3.67 (1H, m, H-6), 2.24–2.12 (1H, m, H-11), 1.85–1.75 (1H, m, H-8 or H-9), 1.74–1.60 (2H, m, H-8 or H-9, H-10), 1.29–1.11 (3H, m, H-8, H-9, H-11), 0.82, 0.79 (6H, s, CH_3_ (*i*Pr)), 0.63 (3H, s, H-12). ^13^C{^1^H} NMR (CDCl_3_, 100 MHz) *δ* 164.8 (C-2), 153.5 (C-4), 136.9, 135.9, 130.0, 124.9, 120.5 (C-3, C_arom_), 102.2 (C-5), 88.3 (C-6), 49.6 (C-13), 47.8 (C-7), 44.9 (C-10), 36.9 (C-11), 28.1, 26.6 (C-8, C-9), 19.7, 18.8 (CH_3_ (*i*Pr)), 13.6 (C-12). HRMS (ESI) *m/z* 435.0566 (calcd for C_20_H_22_Cl_2_NaO_3_S, 435.0559).

***5(S)-3-Chloro-4-[(4-chlorophenyl)sulfonyl]-5-[(1S,2R,4S)-1,7,7-trimethylbicyclo[2.2.1]-heptan-2-yloxy]-2(5H)-furanone (F131)***. Thioether **3** (0.29 g, 0.7 mmol) was dissolved in 10 mL of glacial acetic acid, 33% hydrogen peroxide (0.74 mL, 7.3 mmol) was added under stirring, and the mixture was stirred for 5 days at room temperature (monitored by TLC). When the reaction was complete, the mixture was evaporated to dryness, and the white solid residue was recrystallized from hexane to give sulfone **F131**. Colorless crystals (0.22 g, 70%); mp 142 °C; *R_f_* 0.49; [α]${}_{D}^{20}$ = +158.6 (*c* 1.0, CHCl_3_). IR (ATR) ν_max_ 2991, 2956, 2931, 2889 (C–H), 1786 (C=O), 1622 (C=C_lactone_), 1579, 1475 (C=C_arom_), 1344 (SO_2_ asym), 1163 (SO_2_ sym) cm^–1^. ^1^H NMR (CDCl_3_, 400 MHz) *δ* 8.03, 7.60 (4H, AA′BB′, *N* = ^3^*J*_AB_ + ^5^*J*_AB′_ = 8.6 Hz, H_arom_), 6.23 (1H, s, H-5), 4.16–4.07 (1H, m, H-6), 2.33–2.22 (1H, m, H-11), 1.78–1.63 (3H, m, H-8, H-9, H-10), 1.36–1.23 (1H, m, H-8 or H-9), 1.23–1.05 (2H, m, H-8 or H-9, H-11), 1.04 (3H, s, H-12), 0.89, 0.88 (6H, s, CH_3_ (*i*Pr)). ^13^C{^1^H} NMR (CDCl_3_, 100 MHz) *δ* 162.8 (C-2), 150.6 (C-4), 142.5, 137.0, 134.1, 130.3, 130.0 (C-3, C_arom_), 102.7 (C-5), 90.7 (C-6), 49.9 (C-13), 47.9 (C-7), 44.9 (C-10), 36.8 (C-11), 28.2, 26.7 (C-8, C-9), 19.7, 18.9 (CH_3_ (*i*Pr)), 13.9 (C-12). HRMS (ESI) *m/z* 467.0449 (calcd for C_20_H_22_Cl_2_NaO_5_S, 467.0457).

### 2.3. Antimicrobials

Amikacin, gentamicin, benzalkonium chloride, terbinafine and fluconazole were purchased from Sigma, (St. Louis, MO, USA). Solutions were prepared at a concentration of 20 mg/mL in deionized water, or in dimethyl sulfoxide (DMSO) for fluconazole. Solutions of **F131** were prepared at a concentration of 10 mg/mL in DMSO.

### 2.4. Strains and Growth Conditions

The antimicrobial activity of the compounds was assessed on methicillin-susceptible *Staphylococcus aureus* (MSSA) ATCC 29213 and 9 clinical isolates of *S. aureus* (3 MSSA and 6 MRSA) provided by the Pharmaceuticals Research Center of Kazan Federal University. Antimycotic activity was assessed on 12 clinical isolates of *Candida albicans* (6 fluconazole-resistant strains, and 6 fluconazole-susceptible strains) obtained from the Kazan Research Institute of Epidemiology and Microbiology. The bacterial strains were stored in 10% (*v*/*v*) glycerol stocks, fungal strains in 50% glycerol stock at −80 °C, and freshly streaked on Luria Agar (LA) plates following by their overnight growth at 37 °C before use. To obtain a mature biofilm, bacterial and fungal cells were grown in basal medium (BM broth) [54] in tissue-culture-treated (TC-treated) plates under static conditions for 48 h at 37 °C. A mannitol salt agar (peptone 10 g, meat extract 1 g, NaCl 75 g, D-mannitol 10 g, agar–agar 12 g in 1.0 L tap water, Oxoid) and Sabouraud’s agar (Difco) were used for differential count of *S. aureus* and *C. albicans*, respectively, from *S. aureus–C. albicans* mixed cultures.

### 2.5. Determination of the Minimal Inhibitory (MIC) and the Minimal Bactericidal/Fungicidal Concentrations (MBC/MFC) of **F131** and Reference Antimicrobials

The minimum inhibitory concentration (MIC) was determined by serial 2-fold microdilution in 96-well plates according to the EUCAST rules for antimicrobial susceptibility testing [61] with some modifications. The maximal final concentration of each tested compound was 512 µg/mL. The next well contained two-fold decreasing concentration of the antimicrobial in the range of 0.5–512 μg/mL. The wells were seeded with microbial culture with a final density of 10^6^ CFU/mL in a BM broth [54] as universal medium for all further experiments. The plates were incubated for 24 hours at 37 °C without shaking. The minimal inhibitory concentration of compounds was defined as the concentration that ensures complete suppression of the visible growth of the strains. The minimal bactericidal/fungicidal concentration (MBC/MFC) was determined by inoculation in 3 mL of nutrient broth with 3 μL of culture fluid from wells in which no visible growth was observed. MBC/MFC was considered as the minimum concentration of the compound, which ensures the complete absence of microorganisms growth.

### 2.6. Analysis of the Biofilm–Prevention Concentration (BPC)

To determine the minimal biofilm-prevention concentration, bacterial and fungal cells were grown in 96-well adhesive plastic plates for 48 hours without shaking at 37 °C in 200 μL BM broth with an initial density 3 × 10^7^ CFU/mL in the presence of the test compounds with concentration in the range of 0.5–512 μg/mL. Next, wells were washed with water and subjected to a staining with crystal violet as described in [62]. The minimal biofilm inhibitory concentration was defined as the lowest concentration at which the residual biofilm was two-fold less compared to untreated wells.

### 2.7. Assessment of Synergy between **F131** and Conventional Antimicrobials

To assess a synergy between **F131** and antimicrobials, a checkerboard assay was performed as described previously [41]. Each plate contained serial dilutions of a **F131** and various antimicrobials in a checkerboard pattern. One of the antimicrobial substances [A] was diluted horizontally, and the **F131** [B] vertically on a 96-well plate. The extreme lines and columns contained only one of the considered compounds to determine their MICs in each experiment. The initial concentration of each of the studied antimicrobial agents was 2 × MIC. The plates were incubated at 37 °C for 24 h. The experiments were performed in a triplicate, a growth control without the addition of any antimicrobial agent was included in each plate. The fractional inhibitory concentration index (FICI) for each double combination was calculated as follows:

$$\mathrm{FICI}\;=\;\frac{\mathrm{MIC}\left[A\right]\;\mathrm{in}\;\mathrm{combination}}{\mathrm{MIC}\;\left[A\right]}+\frac{\mathrm{MIC}\left[B\right]\;\mathrm{in}\;\mathrm{combination}}{\mathrm{MIC}\left[B\right]}$$

Interpretation of the obtained FICI values was carried out according to Odds [63]; FICI ≤ 0.5 corresponded to synergy, 0.5 < FICI ≤ 4 an additive effect, while FICI > 4 corresponded to antagonism.

### 2.8. Quantification of Viable Cells

To assess the viability of detached cells and cells in the biofilm in the presence of antimicrobials, a drop plate assay [64] with modifications was used. Briefly, a series of ten-fold dilutions of cell suspension from each well were prepared in 3 technical repeats and 5 μL from each dilution were dropped onto both mannitol salt agar and Sabouraud agar with ciprofloxacin (10 μg/mL) to differentiate *S. aureus* and *C. albicans* cells, respectively. CFUs were counted from the two last drops where 5–15 colonies were grown and averaged. In the case of the biofilm-embedded cells, the wells were pre-washed twice with sterile 0.9% NaCl and biofilms were suspended in 0.9% NaCl by scratching the well bottoms. Additionally, the wells were sonicated for 2 min to disintegrate the remained cell clumps [65]. The dilutions of obtained suspension were prepared and CFUs were counted as described above.

### 2.9. Confocal laser Scanning Microscopy (CLSM)

To assess the localization of **F131** in bacterial and fungal cells, confocal laser scanning microscopy was performed using an Olympus IX83 inverted microscope supplemented with a STEDYCON ultrawide extension platform. Both monospecies and bacterial-fungal cultures of *S. aureus* and *C. albicans* were grown on cell imaging cover slips (Eppendorf) for 48 h in BM broth. The fluorescent derivative of 2(5*H*)-furanone **F145** [53] was added to the cells at a final concentration of 20 μg/mL and biofilms were analyzed at blue (405/410–508 nm) channel.

### 2.10. RNA Isolation and Real-Time One-Step qRT-PCR

To analyze the transcription of the efflux system genes *MDR1, CDR1*, and *CDR2,* the PCR was performed using PCR amplifier "BioRad CFX96" (BioRad, Singapore) using Extra Mix for reverse transcription and quantitative real-time PCR in a one-step method (BioLabMix, Novosibirsk, Russia) with SYBR Blue under conditions recommended by the manufacturer. The oligonucleotides used for the qRT-PCR are shown in Table 1 [66,67,68]. A total RNA was extracted from planktonic *C. albicans* 688 cells subjected to 48-h treatment with either fluconazole or **F131**. Reaction mixture (50 µL) contained 1× RT-qPCR SYBR Blue buffer, 0.1 µM of each primers, 0.1 μM of each dNTPs, 2.5% DMSO, 5% RT-qPCR extra-mix and nuclease-free water (DEPC). The RT–PCR program included reverse transcription at 45 °C for 30 min, followed by 37 cycles of primer annealing–elongation–melting. Primer annealing temperatures were calculated using the Tm Calculator service (https://tmcalculator.neb.com, accessed on 6 January 2022). The elongation time was calculated based on the size of the synthesized fragment and the speed of the DNA polymerase. Real-time PCR monitoring was carried out at the stage of final synthesis at 72 °C. The 18s rRNA and GAPDH genes were used as references; the transcription level of qCDR1, qCDR1 and MDR1 genes was normalized by the 18s rRNA transcription level.

### 2.11. Data Analysis

All experiments were performed in three biological replicates with three technical replicates. The data was analyzed using GraphPad Prism version 6.0 for Windows (GraphPad Software, San Diego, CA, USA). The significance of difference has been checked by using the Kruskal–Wallis test. Significant differences from control were considered at *p* < 0.05.

## 3. Results

### 3.1. Synthesis of Furanone **F131**

**Figure 1:**
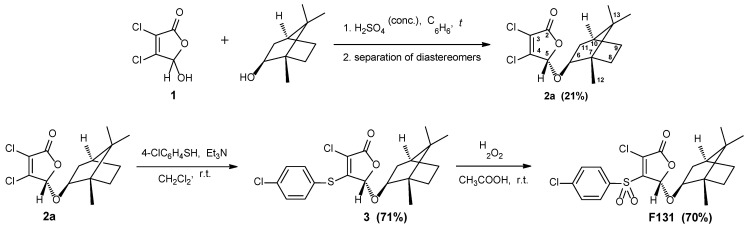
Synthesis of 2(5*H*)-furanone derivative **F131**.

Synthesis of novel 2(5*H*)-furanone derivative **F131** possessing sulfonyl group and *l*-borneol moiety was achieved via three steps from commercially available mucochloric acid **1** (Figure 1). Following a previously published procedure, mucochloric acid **1** was first converted to the corresponding 5-(*l*-bornyloxy) derivative, obtained as a mixture of two diastereomers **2a** + **2b** [52,60]. The pure stereoisomer **2a** with *S*-configuration of the carbon atom C^5^ of the γ-lactone cycle was isolated after two recrystallizations from hexane.

**Figure A1:**
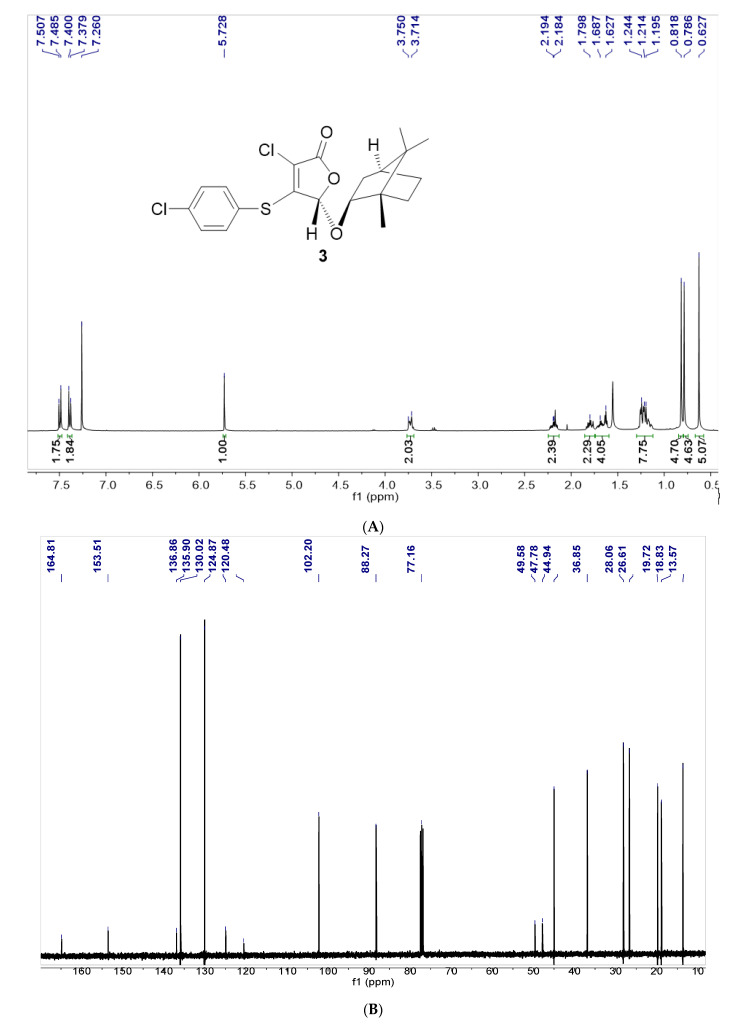
^1^H (**A**) and ^13^C{^1^H} (**B**) NMR spectra of compound **3** (CDCl_3_).

**Figure A2:**
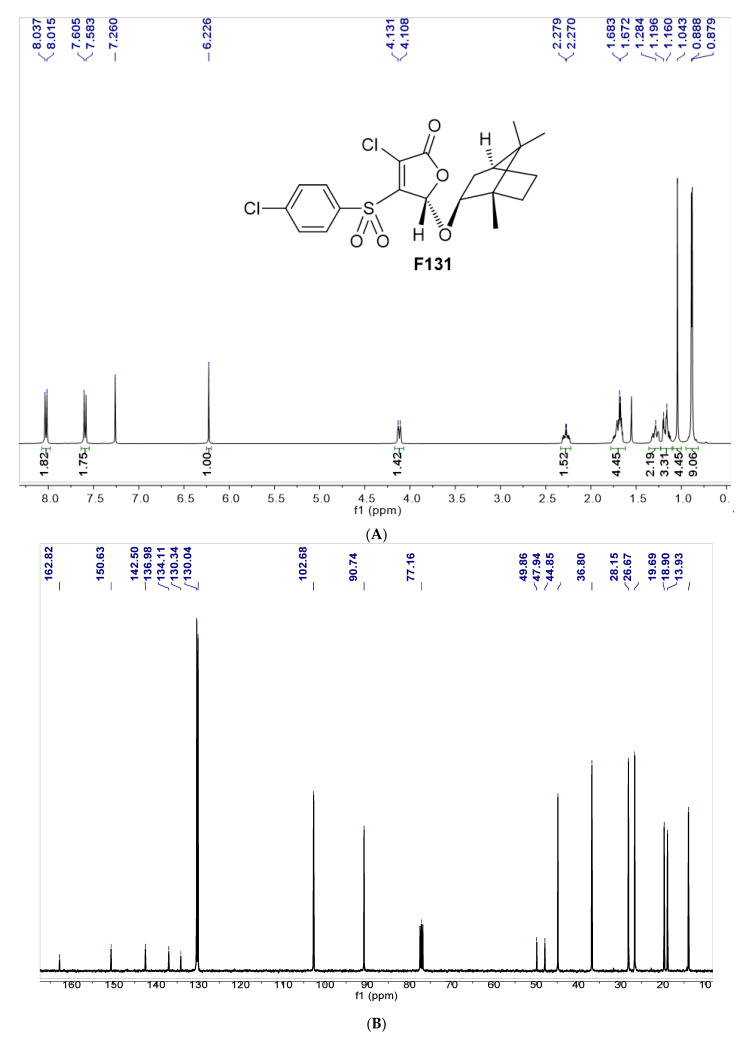
^1^H (**A**) and ^13^C{^1^H} (**B**) NMR spectra of compound **F131** (CDCl_3_).

**Figure A3:**
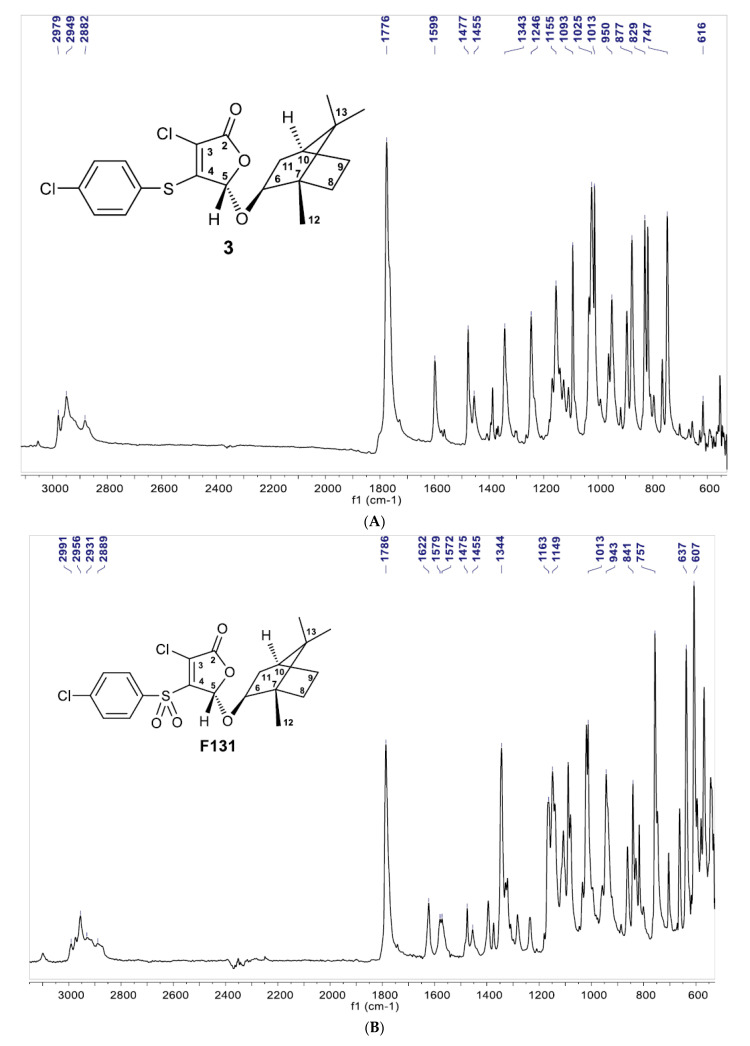
IR spectra of thioether **3** (**A**) and sulfone **F131** (**B**).

**Figure A4:**
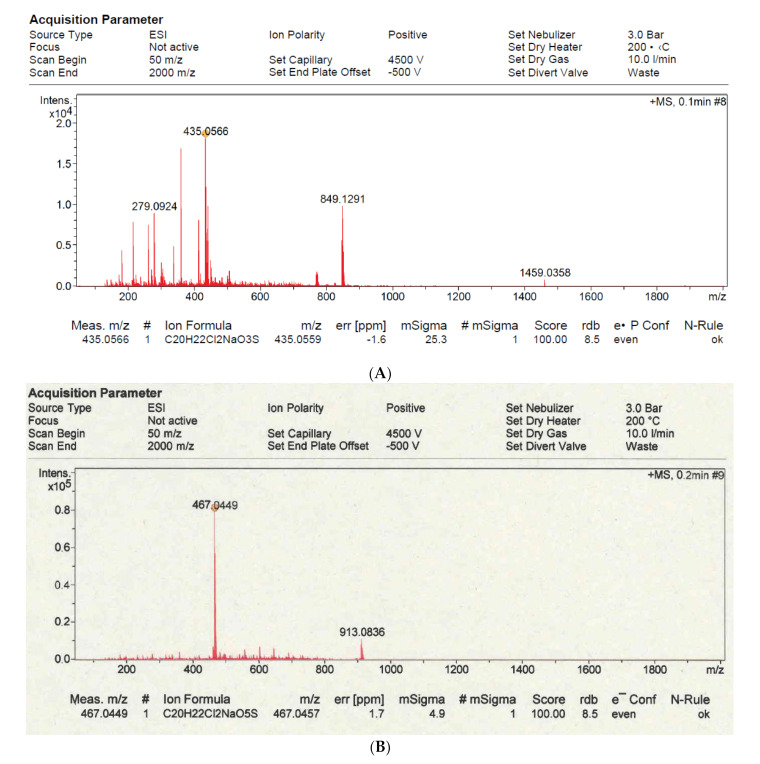
Mass spectra of thioether **3** (**A**) and sulfone **F131** (**B**).

Next, regioselective introduction of 4-chlorothiophenol fragment into the molecule of the isolated stereoisomer **2a** was carried out. It is well-known that interaction of 3,4-dihaloderivatives of 2(5*H*)-furanone with different thiols in the presence of basic compounds goes with the substitution of halogen atom in the fourth position of the lactone cycle [69,70]. The thiilation reaction was performed in dichloromethane at room temperature using the equimolar ratio of furanone **2a**, thiol and triethylamine. Chiral thioether **3** was obtained with 71% yield and subjected to a further oxidation reaction. We used a simple and convenient method of the synthesis of furanone sulfonyl derivatives, based on the treatment of thioethers with excess of hydrogen peroxide in acetic acid at room temperature [70,71]. Thus, the desired novel sulfone **F131** in a stereomerically pure form was isolated with 70% yield as colorless crystals (Figure 1). The structure of borneol-containing furanones **3** and **F131** has been characterized by IR and NMR spectroscopy (Figure A1, Figure A2, Figure A3 and Figure A4).

### 3.2. Antimicrobial and Biofilm Preventing Activity of **F131**

The antimicrobial and antifungal properties of **F131** were evaluated using a range of *S. aureus* clinical isolates (n = 9) and methicillin-sensitive *S. aureus* ATCC 29213 (Table 2), as well as twelve *C. albicans* clinical isolates with different susceptibility to fluconazole (Table 3). All clinical isolates of *S. aureus* were resistant to gentamicin. MRSA strains were also resistant to amikacin, while MSSA strains remained sensitive to the antibiotic. Six of the twelve clinical isolates of *C. albicans* were resistant to fluconazole. MIC of **F131** ranged within 8–16 µg/mL for *S. aureus* isolates, and 32–128 µg/mL for *C. albicans* isolates (Table 2 and Table 3) suggesting that susceptibility of isolates to **F131** does not correlate with resistance to conventional antimicrobials. Of note, the *l*-borneol itself was ineffective against both *S. aureus* and *C. albicans* (MIC > 1024 for all isolates). The minimal bactericidal concentration (MBC) and the minimal fungicidal concentration (MFC) values of **F131** ranged within 32–128 µg/mL and 128–1024 µg/mL respectively (Table 2 and Table 3). These data indicate a considerable bactericidal and fungicidal activity of **F131**, as the MIC/MBC ratio lies within 2–4, with a few exceptions.

**Figure 2:**
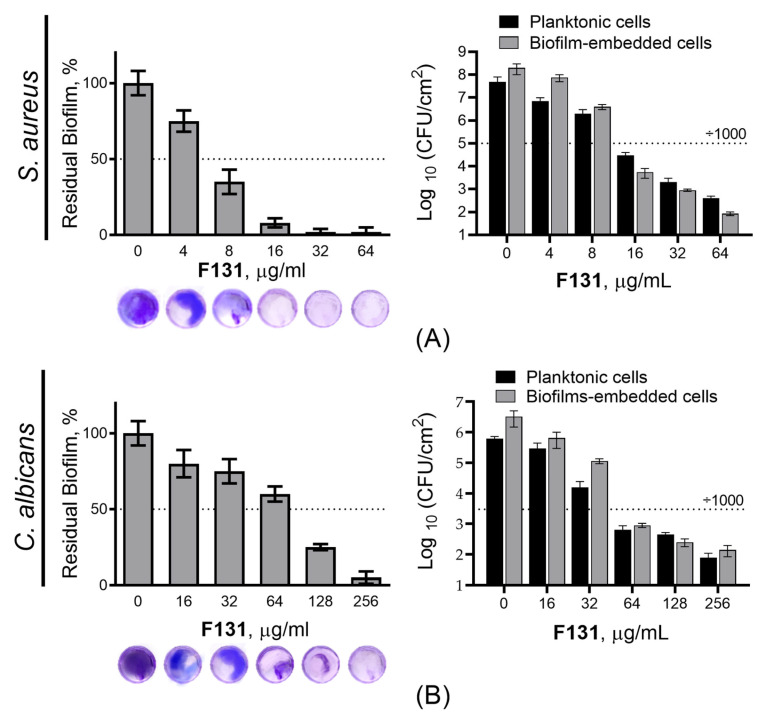
The biofilm-preventing activity (BPC) of **F131** against biofilms of (**A**) *S. aureus* ATCC 29213, and (**B**) *C. albicans* 688^FR^. **F131** was added to the broth and cells were growing for 24 h. Next the biofilms were subjected to crystal-violet staining, the viable planktonic and biofilm-embedded cells were assessed by CFUs counting.

**Figure 3:**
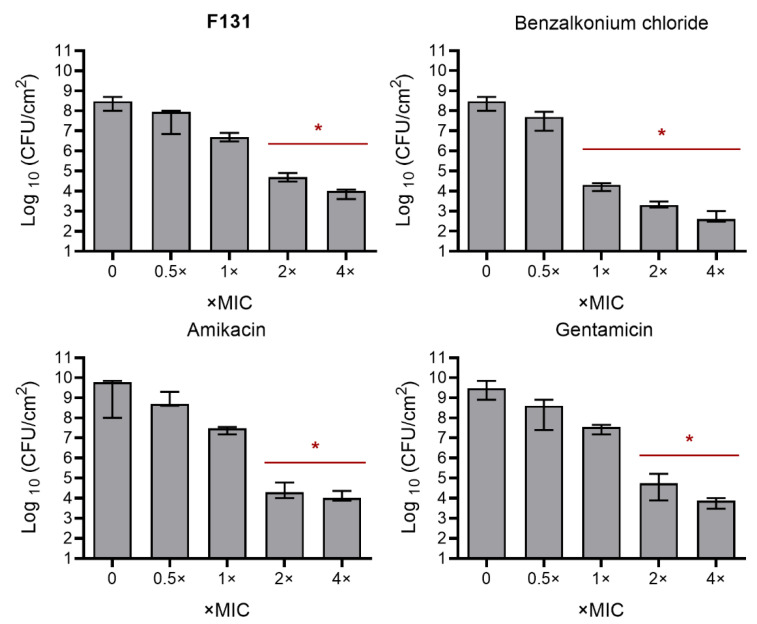
Comparison of antibiofilm activity of antibiotics against *S. aureus* ATCC29213 isolate. *S. aureus* ATCC 29213 isolate was grown for 48 h without agitation in a 96-well flat-bottom plate in the presence of amikacin, gentamicin, benzalkonium and **F131** in concentrations of 0.5-4-fold MIC, which were as follows: 8 mg/L for amikacin, 4 mg/L for gentamicin, 0.5 mg/L for benzalkonium, and 8 mg/L for **F131**. The asterisks (*) denote a statistically significant difference in cells viability in the untreated wells and treated ones in Kruskal–Wallis test (*p* < 0.05).

**Figure 4:**
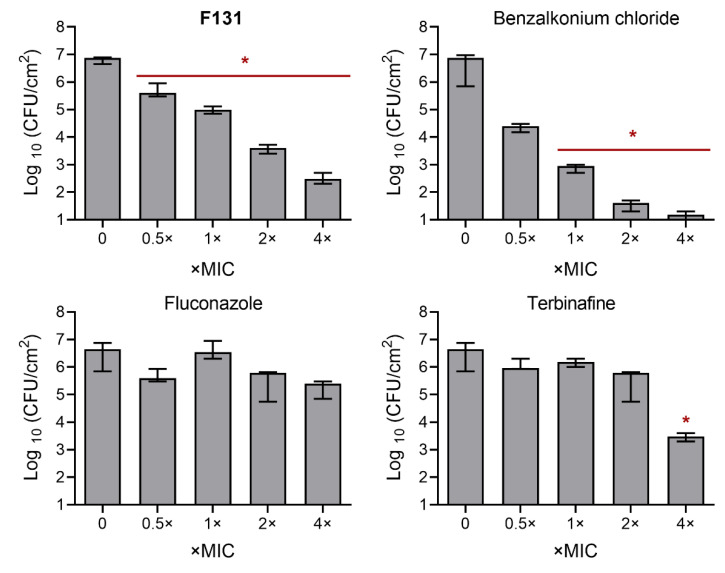
Comparison of antibiofilm activity of antimycotics against *C. albicans* isolate 688^FR^. *C. albicans* isolate was grown for 48 h under static conditions in a 96-well flat-bottom plate in the presence of sub-MICs of fluconazole, terbinafine, benzalkonium and **F131**. The concentrations are given in relative units as X-fold MIC, which were as follows: 1024 mg/L for fluconazole, 128 mg/L for terbinafine, 4 mg/L for benzalkonium, and 64 mg/L for **F131**. The asterisks (*) denote a statistically significant difference in cells viability in the untreated wells and treated ones in Kruskal–Wallis test (*p* < 0.05).

Further, since the biofilm-inhibiting properties for many 2(5*H*)-furanone derivatives have been reported, the ability of **F131** to repress the biofilm formation by *S. aureus* and *C. albicans* was assessed. For that *S. aureus* and *C. albicans* were grown for 48h under static conditions in the presence of **F131**. Then, the biofilm was quantified by both crystal violet staining and direct count of viable cells. The compound led to two-fold reduction of the *S. aureus* biofilm biomass at the concentration of 8 µg/mL, whereas the three-log decrease of viable cells in the biofilm was observed at 16 µg/mL (Figure 2A). For fluconazole-resistant *C. albicans* 688, the biofilm biomass was reduced twice at 128 µg/mL, and 64 µg/mL led to a 1000-fold decrease of viable cell in the biofilm (Figure 2B). Of note, the dose-dependent dynamics of viable cells decrease was similar for both planktonic and adherent (biofilm-forming) ones, suggesting that the observed biofilm prevention activity is driven, apparently, rather by the reduction of viable cells than the true inhibition of the biofilm formation. This hypothesis is also supported by fact; that the conventional antimicrobials such as gentamicin, amikacin, fluconazole, terbinafine and benzalkonium chloride also reduced the amount of viable cells in the biofilm at their respective two-fold MICs (Figure 3 and Figure 4).

### 3.3. Antibiofilm Activity of **F131** on S. aureus and C. albicans Mixed Biofilms

**Figure 5:**
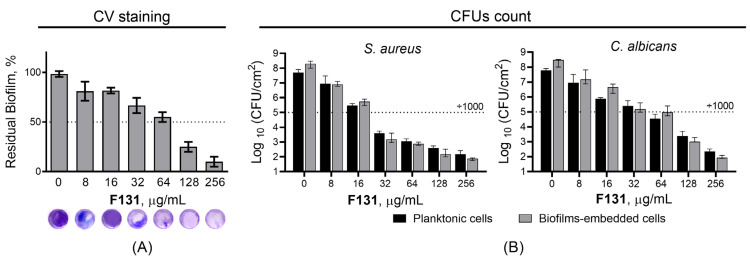
The biofilm-preventing activity (BPC) of **F131** against *S. aureus–C. albicans* mixed biofilms. **F131** was added prior the inoculation with following cell growth for 24 h. The biofilms were quantified after (**A**) crystal violet staining, (**B**) CFU count.

*S. aureus* ATCC 29213 and *C. albicans* 688 fluconazole-resistant isolates were chosen to apply tests on mixed bacterial–fungal biofilms. The *S. aureus*–*C. albicans* mixed culture was grown under static conditions in BM broth in the presence of **F131** in concentrations varying from 2 to 256 µg/mL. After 48h, the biofilms were quantified by the crystal violet staining and CFUs count by plating on mannitol salt agar, and Sabouraud agar with ciprofloxacin (10 μg/mL) to differentiate *S. aureus* and *C. albicans* cells, respectively. The **F131** at 128 μg/mL completely inhibited the biofilms formation in mixed cultures (Figure 5a). The growth of *S. aureus* and *C. albicans* in mixed culture was repressed at 16 µg/mL and 64 µg/mL, respectively (Figure 5b), similarly to monocultures.

**Figure 6:**
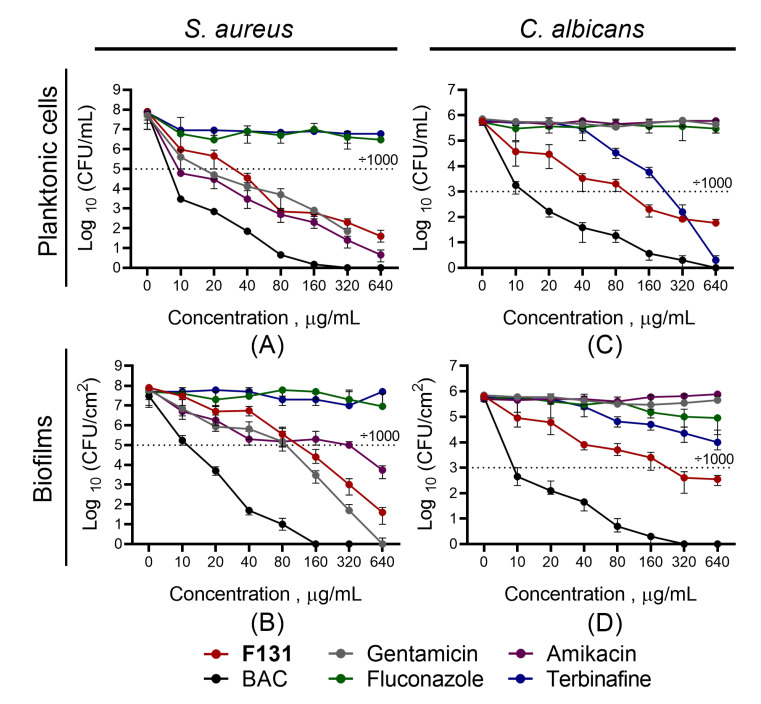
Biofilm eradication activity of **F131** and reference drugs on *S. aureus–C. albicans* mixed biofilms. Different concentrations (5–320 μg/mL) of **F131** or reference antimicrobials and antimycotics were added to the established biofilms. After 24 h, biofilms-viable *S. aureus* and *C. albicans* cells were differentially counted on mannitol salt agar and Sabouraud agar, respectively, with ciprofloxacin (10 μg/mL). Point 0 corresponds to untreated cells (negative control). BAC—benzalkonium chloride. Data are presented as medians from four independent experiments with IQR. Dotted line shows a three-log-decrease of viable cells.

To further analyze whether **F131** not only prevents biofilm formation but also eradicates already established ones, different concentrations (5–320 μg/mL) of **F131** or reference antimicrobials were added to the established 24 h old mixed biofilms. After 24 h, biofilms and detached cells clumps in culture liquid were quantified by differential CFUs counting. For the detached *S. aureus* cell clumps, the efficiency of **F131** was comparable with those of both aminoglycosides. Further, both **F131** and aminoglycosides reduced the CFUs number of *S. aureus* in biofilm by three orders of magnitude at a concentration of 80 μg/mL (2.5–5 × MBC), whereas 10 μg/mL (5 × MBC) of benzalkonium chloride was required to achieve the same effect (Figure 6B). On *C. albicans*, the efficiency of **F131** was comparable with those of terbinafine. Of note, neither terbinafine nor **F131** led to three-order reduction in the CFUs count even at concentrations up to 640 μg/mL (Figure 6D).

**Figure 7:**
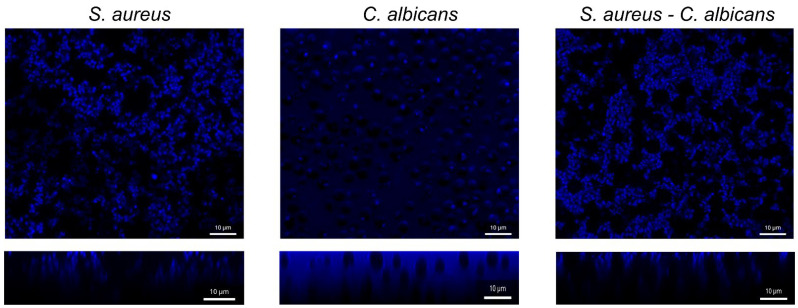
The CLSM-analysis of **F145** penetration into *S. aureus*, *C. albicans* and *S. aureus–C. albicans* mixed biofilms. The images show a plan view on a basal biofilm layer (indicated by *X* and *Y* axis) and a cross section thought the biofilm (*Z* axis). The scale bars indicate 10 µm.

These findings raised questions about the penetration possibility of furanone derivatives into the biofilm matrix formed by *S. aureus–C. albicans*. To confirm these suggestions, the fluorescent analogue of 2(5*H*)-furanone named **F145,** possessing the fluorescent moiety instead of *l*-borneol and described earlier [72], was added to established 24-hours-old mono- and mixed-biofilms of *S. aureus–C. albicans*, and after 20 min, incubation samples were analyzed by confocal laser scanning microscopy. The fluorescence of **F145** was observed throughout the whole biofilm (Figure 7), confirming its fast penetration through the *S. aureus*–*C. albicans* biofilm matrix.

### 3.4. Synergistic Effects of **F131** with Other Antimicrobials on S. aureus–C. albicans Mixed Biofilms

Previously, it has been shown that the 2(5*H*)-furanone derivative **F105** containing *l*-menthol and sulfonyl moieties, exhibits a synergy with aminoglycosides and benzalkonium chloride against *S. aureus* [41] and potentiates the antifungal activity of fluconazole and terbinafine against *C. albicans* cells [53]. Taking into account the similar structures of compounds **F105** and **F131**, which are both chiral furanone sulfones, possessing a fragment of terpene alcohol in the fifth position of the lactone cycle, we assumed similar properties for the latter.

Therefore, the synergism of **F131** in combination with conventional antimicrobials was analyzed by the checkerboard assay on *S. aureus* ATCC 29213 and *C. albicans* 688 fluconazole-resistant isolate. The values of the fractional inhibitory concentrations (FICI) were determined in checkerboard assay and the median was calculated. For bacterial and fungal mono-species cultures, **F131** demonstrated marked synergism with gentamicin with FICI of 0.1875, and fluconazole with FICI of 0.375. Additionally, pronounced synergy was observed also for benzalkonium chloride for *S. aureus* (FICI = 0.25) and *C. albicans* (FICI = 0.1875) (Table 4 and Table 5). Thus, the presence of **F131** significantly lowered the MIC of gentamicin by 16-fold, and the MIC of benzalkonium chloride by eight-fold. Of note, the presence of **F131** altered fluconazole susceptibility in fluconazole-resistant isolate (*C. albicans* 688), as it became more sensitive to fluconazole with FIC value of 128 (Table 5). In turn, FIC values of **F131** decreased up to eight-fold when combining with gentamicin, fluconazole, and benzalkonium chloride.

**Figure 8:**
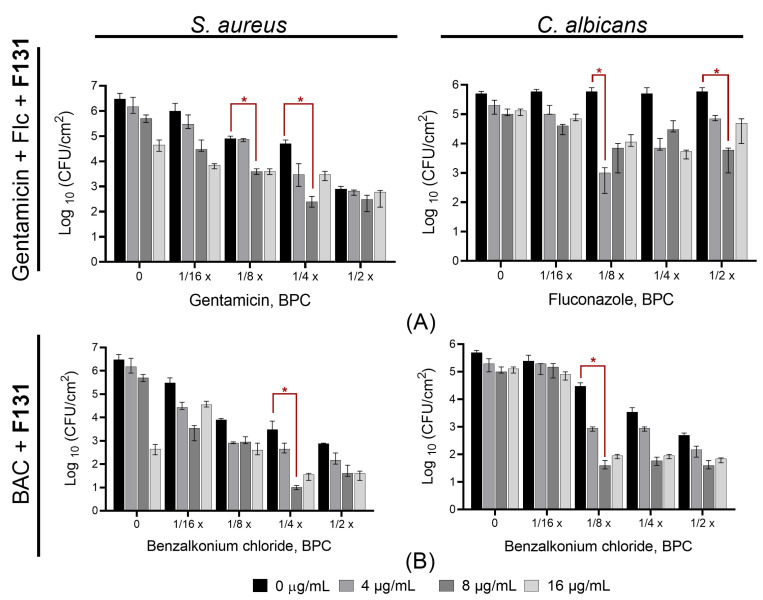
Quantification of the synergistic effects of **F131** on mixed *S. aureus*–*C. albicans* biofilm formation. Three concentrations of **F131** (4, 8, 16 µg/mL) were combined with sub-BPC concentrations of either benzalkonium chloride (BAC) or gentamicin + fluconazole (Flc) (in 1:1 ratio of respective BPC). The viable cell counts were determined by CFU counting by plating on mannitol salt agar and Sabouraud agar with ciprofloxacin (10 μg/mL) to differentiate *S. aureus* and *C. albicans* cells, respectively. The experiments were performed in triplicate and the median and error are shown. The asterisks (*) denote a statistically significant difference in cells viability in the untreated wells and treated ones in Kruskal–Wallis test (*p* < 0.05).

Further, we questioned whether the synergistic effect of 2(5*H*)-furanone derivatives could be used to eradicate mixed *S. aureus–C. albicans* infection. Therefore, we tested the effect of combination of **F131** with conventional antimicrobials on mixed *S. aureus–C. albicans* biofilms formation and eradication, since the biofilm-embedded consortium became extremely resistant to treatment compared to planktonic cells. Firstly, the *S. aureus–C. albicans* mixed cultures were inoculated in BM broth in the presence of various concentrations of antimicrobials as indicated. **F131** concentrations were 4, 8, and 16 μg/mL, which correspond to ¼–1.0 BPCs for *S. aureus* and 1/16–1/4 BPCs for *C. albicans*. In bacterial–fungal mixed biofilms, a considerable synergism between **F131** and gentamicin–fluconazole mix was detected at 1/4 BPC of gentamicin and 1/8 BPC of fluconazole against *S. aureus–C. albicans*, as judged from the observed three-order reduction of the viable cell number per cm^2^ for the untreated control (Figure 8A). Moreover, the combination of **F131** with sub-BPCs of benzalkonium chloride (1/4 BPC for *S. aureus* and 1/8 BPC for *C. albicans*) decreased the viable cell number in mixed biofilms by three orders of magnitude, indicating a strong synergistic effect (Figure 8B).

**Figure 9:**
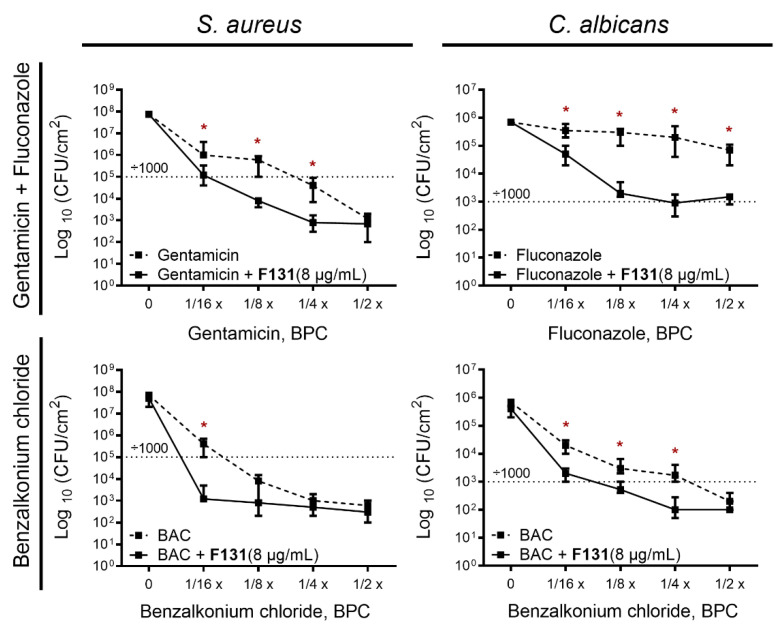
Antifungal and antibacterial effect of fluconazole–gentamicin mixture and benzalkonium chloride alone and in combination with 8 µg/mL **F131** against *S. aureus*–*C. albicans* mixed biofilms. Sub-BPCs of fluconazole–gentamicin mixture or benzalkonium chloride were added prior the inoculation with following cell growth for 48 h. After 24 h, biofilms-viable *S. aureus* and *C. albicans* cells in biofilms were differentially counted on mannitol salt agar and Sabouraud agar with ciprofloxacin (10 μg/mL), respectively. Point 0 corresponds to untreated cells (negative control). BAC—benzalkonium chloride. Data are presented as medians from four independent experiments with IQR. Dotted line shows three-log-decrease of viable cells count. (*) denote a statistically significant difference in cells viability in the untreated wells and treated ones in Kruskal–Wallis test (*p* < 0.05).

For convenience, we plotted a graph representing the antifungal and antibacterial effect of fluconazole–gentamicin mix and benzalkonium chloride alone and in combination with fixed concentration of **F131** (8 µg/mL) against *S. aureus*–*C. albicans* mixed biofilms (Figure 9). As could be seen from the Figure 9, in the presence of 8 µg/mL **F131**, the concentrations of fluconazole, gentamicin and benzalkonium chloride, required to decreases CFUs number by three orders of magnitude are four-fold lower when compared with solely antimicrobials. Taken together, these data clearly indicate that **F131** at a concentration of 8 µg/mL (1/2 BPC for *S. aureus,* and 1/8 BPC for *C. albicans*) is able to significantly reduce the number of viable cells when combining with sub-BPCs of conventional antifungals and antibacterial drugs.

### 3.5. The Combination of **F131** with Fluconazole Significantly Decreased the Expression Level of CDR1 and CDR2 Genes

**Figure 10:**
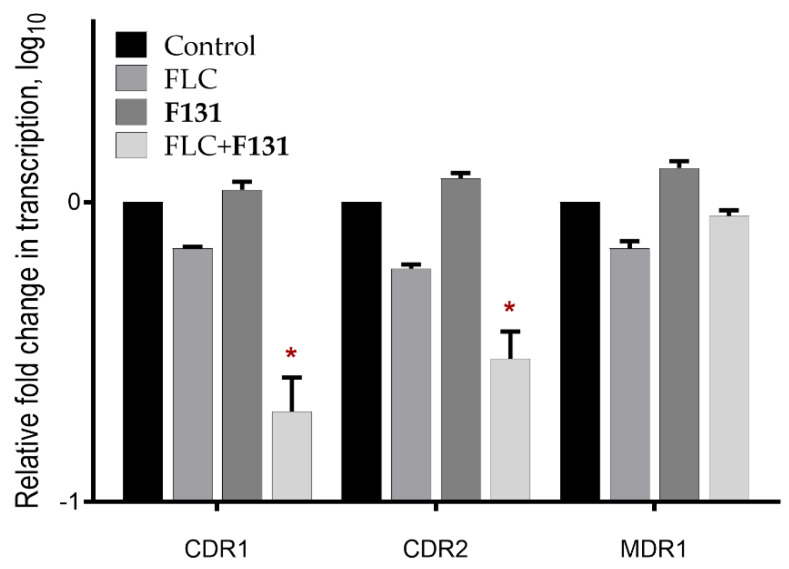
Relative expression levels of efflux system genes CDR1, CDR2 and MDR1 in fluconazole-resistant mixed culture *C. albicans* and *S. aureus*. Cells were treated with fluconazole (FLC) (32 μg/mL), **F131** (8 μg/mL) and their combination, respectively. Data normalized for control group. The 18s rRNA gene was used as reference. (*) denote a statistically significant difference in cells viability in the untreated wells and treated ones in Kruskal–Wallis test (*p* < 0.05).

The mRNA levels of the efflux system genes *MDR1, CDR1*, and *CDR2* were analyzed by quantitative real-time RT-PCR in *C. albicans* 688 isolate (resistant isolate). Cells were grown in the presence of 32 µg/mL fluconazole and/or 8 µg/mL **F131** for 24 hours. The same volume of solvents (DMSO and ethanol) was added to the untreated control group of cells. The results showed that **F131** solely did not affect the expression of the mentioned genes, since the coefficient of relative expression of *MDR1*, *CDR1* and *CDR2* were 1.12, 1.29 and 1.31, respectively. When treating with fluconazole, the expression level was suppressed of the *MDR1*, *CDR1* and *CDR2* genes, although underestimated (relative expression 0.71, 0.62 and 0.69, respectively). It is noteworthy that the expression of *CDR1* and *CDR2* genes decreased significantly after the combined treatment with fluconazole and **F131** (relative expression 0.22 and 0.31, respectively) (Figure 10).

## 4. Discussion

Taking into account the high mortality and morbidity rates associated with *S. aureus*–*C. albicans* mixed infections, there is an always pursuit to find new potential alternatives that ensure better efficiency and lower toxicity during the management of candida mixed infections, especially in the course of strains that are resistant to fluconazole. Here, we show that the novel 2(5*H*)-furanone derivative **F131,** consisting of two pharmacophores (2(5*H*)-furanone cycle and *l*-borneol moiety), exhibits an antimicrobial and biofilm-preventing activity as well as potentiates conventional antibiotics and antifungal agents against *S. aureus* and *C. albicans* and their mixed culture. Earlier, we showed that the structural analogue of **F131** with *l*-menthol fragment (3-chloro-5(*S*)-[(1*R*,2*S*,5*R*)-2-isopropyl-5-methylcyclohexyloxy]-4-[4-methylphenylsulfonyl]-2(5*H*)-furanone **F105**) exhibited comparable antibacterial and antifungal activity against *S. aureus* [41] and *C. albicans* [53]. **F131** possesses the *l*-borneol instead of *l*-menthol fragment and demonstrates higher antimicrobial activity. Interestingly, many previous studies reported the anti-inflammatory and antimicrobial activity of borneol derivatives [73,74,75]. Some aromatic borneol compounds were significantly active with MIC_50_ = 125 µg/mL for both *S. aureus* and *C. albicans* [76]. In our previous study we examined the relationship between the chemotype and antimicrobial activity of 2(5*H*) derivatives. We found that the *l*-borneol moiety is an essential part of the molecule of 3,4-dichloro-5(*S*)-[(1*S*,2*R*,4*S*)-1,7,7-trimethylbicyclo[2.2.1]heptan-2-yloxy]-2(5*H*)-furanone (**F123**), with respect to demonstrating its antibacterial activity against gram-positive bacterium *B. cereus* [52]. This data raised a question whether the borneol fragment is responsible for the observed antimicrobial activity of **F131.** For that, we tested the activity of terpene alcohol *l*-borneol against both *S. aureus* and *C. albicans* in mono and mixed cultures. However, our data showed that *l*-borneol itself is not able to inhibit the growth of either *S. aureus* or *C. albicans*, suggesting that this terpene alcohol apparently facilitates the penetration of the multipharmacophore drug into both the cells, probably thereby also providing the synergy with other antimicrobials (Table 2 and Table 3), and into biofilms (Figure 7) thus exhibiting biofilm-eradicating activity (Figure 6).

Our data show that **F131** demonstrates bactericidal and fungicidal activity, as the MIC/MBC ratio lies within 2–4, with a few exceptions (Table 2 and Table 3). Moreover, the susceptibility of isolates to **F131** does not correlate with resistance to conventional antimicrobials, making this compound suitable to the treatment of infections caused by resistant strains, including mixed *C. albicans*–*S. aureus* infections. In addition, **F131** was able to prevent the biofilm formation by both pathogens, either separately or in the mixed culture (Figure 2 and Figure 5). Generally, BPCs of **F131** were found to approximately correspond to MICs, suggesting indirect biofilm-preventing activity of **F131**, apparently because of the reduction of viable cells rather than the true inhibition of the biofilm formation. Additionally, this assumption is supported by noting that the amount of viable planktonic cells also decreased by three orders of magnitude at the corresponding BPCs of **F131** against both *S. aureus* and *C. albicans* (Figure 2). Thus, it could be suggested that the biofilm suppression was rather the consequence of cell growth repression. Interestingly, for *C. albicans* the BPCs were a little higher than MICs. This phenomenon might be attributed to the induction of biofilm formation by the fungi under sub-lethal concentrations of furanone derivatives [53]. Having a closer look at the **F131** chemotype, **F131** is a halogenated furanone containing a chlorine atom in the third position of a five-membered cycle, but our findings are not in agreement with previous studies that reported a direct biofilm-inhibiting activity of some halogenated furanones against gram-positive bacteria such as Bacilli and Staphylococci [48,54].

## 5. Conclusions

Taken together, our in vitro data allows the assumption of the **F131** chemotype as a biofilm-preventing agent, especially in the course of skin infections caused by *Candida*–*Staphylococcus* mixed biofilms. In addition, due to its synergistic effect with conventional antimicrobials, the combination of the latter with **F131** or **F131**-like compounds would give rise to the minimization of the required therapeutic doses and thus reduction of side effects, toxicity and tolerance development by pathogens. Nevertheless, further in vitro assays for the biosafety of **F131** and in vivo studies are required to understand further perspectives to managing *Candida*–*Staphylococcus* mixed infections. 

## Figures and Tables

**Table 1:** The oligonucleotides used for the qRT-PCR.

Name	Sequence
q18s for	5’ GGATTTACTGAAGACTAACTACTG 3’
q18s rev	5’ GAACAACAACCGATCCCTAGT 3’
qGAPDH for	5’ GTCTCCTCTGACTTCAACAGCG 3’
qGAPDH rev	5’ ACCACCCTGTTGCTGTAGCCAA 3’
qCDR1 for	5’ GTACTATCCATCAACCATCAGCACTT 3’
qCDR1 rev	5’ GCCGTTCTTCCACCTTTTTGTA 3’
qCDR1 for	5’ TGCTGAACCGACAGACTCAGTT 3’
qCDR2 rev	5’ AAGAGATTGCCAATTGTCCCATA 3’
qMDR1 for	5’ TCAGTCCGATGTCAGAAAATGC 3’
qMDR2 rev	5’ GCAGTGGGAATTTGTAGTATGACAA 3’

**Table 2:** Susceptibility of *S. aureus* to **F131,** gentamicin, amikacin and benzalkonium chloride. MIC and MBC values are shown in µg/mL.

*S. aureus* Strains and Isolates	MIC, µg/mL				MBC, µg/mL			
	F131	Gen (** Ecoff = 2)	Ami (** Ecoff = 16)	BAC *	F131	Gen	Ami	BAC *
ATCC 29213 (MSSA)	8	4	8	0.5	32	16	32	2
18 (MSSA)	8	4	8	0.5	32	16	32	2
25 (MSSA)	8	8	8	0.5	64	32	32	2
26 (MSSA)	16	8	8	0.5	32	32	32	4
1053 (MRSA)	8	16	32	0.5	64	32	32	4
1065 (MRSA)	8	4	32	0.5	32	32	128	2
1130 (MRSA)	8	8	32	0.25	64	16	256	4
1145 (MRSA)	16	4	64	0.25	32	32	256	4
1167 (MRSA)	8	32	32	0.5	32	64	256	2
1168 (MRSA)	16	16	32	0.25	128	16	128	4

* BAC—benzalkonium chloride, Gen—gentamicin, Ami—amikacin; ** Ecoff—epidemiological cut-off value; the MIC value is above the ECOFF defines that the bacterium is likely to be resistant [61].

**Table 3:** Susceptibility of *C. albicans* clinical isolates to **F131**, fluconazole, terbinafine and benzalkonium chloride. MIC and MBC values are shown in µg/mL.

*C. albicans* Isolates	MIC				MFC			
	F131	FLC	TRB	BAC	F131	FLC	TRB	BAC
K4940 FR	64	>512	128	4	>512	>512	256	4
K5050 FR	64	>512	128	4	>512	>512	256	4
K4074 FR	64	>512	256	4	256	>512	512	4
K3957 FR	64	>512	128	4	256	>512	512	4
661FR	32	>512	256	2	256	>512	512	4
688 FR	64	>512	128	2	128	>512	256	4
701	128	16	128	2	128	>512	512	4
703	32	16	128	4	256	>512	256	8
761	32	64	256	2	256	>512	512	8
762	64	32	128	2	256	>512	256	4
722	32	128	64	4	128	>512	256	4
748	64	128	128	2	128	>512	256	4

FLC—fluconazole, TRB—terbinafine, BAC—benzalkonium chloride.

**Table 4:** Biofilm-prevention concentration, fractional inhibitory concentration and FICI values of **F131** on *S. aureus* ATCC 21239 biofilms.

	BPC	BPC (F131)	FIC	FIC (F131)	FICI_min_
Gentamicin	16	16	1	2	0.1875
Amikacin	16	16	8	4	0.75
Benzalkonium chloride	0.5	16	0.0625	2	0.25

BPC—biofilm-prevention concentration, FIC—fractional inhibitory concentration.

**Table 5:** Biofilm-prevention concentration, fractional inhibitory concentration and FICI values of **F131** on *C. albicans* 688^FR^ biofilms.

	BPC	BPC (F131)	FIC	FIC (F131)	FICI_min_
Fluconazole	512<	128	128	16	0.375
Terbinafine	512	128	256	64	1
Benzalkonium chloride	4	128	0.5	8	0.1875

BPC—Biofilm-prevention concentration, FIC—fractional inhibitory concentration.

## Data Availability

All data are included in the manuscript.
